# Draft genome sequences of antibiotic-resistant *Serratia* and *Enterobacter* species isolated from imported fresh produce in Georgia, USA

**DOI:** 10.1128/mra.01139-23

**Published:** 2024-05-02

**Authors:** Issmat I. Kassem, Jiayue Wang, Anahita Gorbani Tajani, Malak A. Esseili, Jouman Hassan, Iman Yassine, Marwan Osman, Bledar Bisha

**Affiliations:** 1 Department of Food Science and Technology, Center for Food Safety, University of Georgia, Griffin, Georgia, USA; 2 Department of Animal Science, University of Wyoming, Laramie, Wyoming, USA; 3 Nuffield Department of Population Health, University of Oxford, Oxford, United Kingdom; 4 Department of Neurosurgery, Yale University School of Medicine, New Haven, Connecticut, USA; University of Maryland School of Medicine, Baltimore, Maryland, USA

**Keywords:** antibiotic resistance, fresh produce, *Serratia*, imported food, *Enterobacter*

## Abstract

Imported foods play an essential role in food security and in fulfilling consumer demand. However, these foods can also carry antibiotic-resistant bacteria, which might be introduced into the country of importation. Here, we report the draft genomes of antibiotic-resistant bacteria that were isolated from imported fresh produce in Georgia, USA.

## ANNOUNCEMENT

In 2021, approximately 38% of the fresh vegetables available in the USA were imported from other countries, primarily Mexico and Canada (1, 2). While imported foods are monitored and required to be safe, they can carry antibiotic-resistant (ABR) non-pathogenic bacteria (3, 4) that are not normally targeted in food safety management systems. Subsequently, food imports can potentially spread ABR bacteria that can act as reservoirs for the dissemination of antibiotic resistance determinants between countries (3, 4). Here, we performed whole-genome sequencing (WGS) analysis of ABR bacteria that were isolated from fresh produce imported into the USA.

Forty imported fresh produce samples were collected from four major retail grocery stores in Georgia, USA. The samples were aseptically transferred to the laboratory on ice and processed within 2 h of collection. The samples were suspended in 100 mL of sterile buffered peptone water (Oxoid, UK) in a stomacher bag and gently massaged for 1 minute. An aliquot (100 µL) of the mixture was spread onto Rapid’*E.coli* 2 agar (BioRad, USA) plates, which were incubated at 37°C for 24 h under aerobic conditions. Colonies were selected randomly and purified further on Rapid’*E.coli* 2 agar. Antibiotic susceptibility patterns were determined using the Kirby-Bauer disk diffusion assay (5). The identity of the isolates was determined using matrix-assisted laser desorption/ionization time-of-flight (MALDI-TOF) mass spectrometry with the Bruker Ultraflex II instrument (Bruker, USA) and the Bruker Biotyper RTC software (v.3.1) (6). Isolates were selected for WGS analysis based on their ABR phenotype and identity.

Pure colonies of each isolate were maintained on Brain Heart Infusion Agar (Difco, USA) plates that were incubated at 37°C for 18 h. Colonies were transferred with inoculation loops and suspended in the buffer supplied in the QIAamp DNA minikit (Qiagen, USA) to isolate genomic DNA as described in the manufacturer’s protocols. The DNA was quantified using the Qubit double-stranded DNA (dsDNA) broad-range assay kit (Invitrogen, USA) (7–9). Libraries were prepared using NEBNext Ultra DNA Library Prep Kit (NEB, USA) and the NEBNext Multiplex Oligos for Illumina (96 Index Primers). Library concentrations were assessed using the Qubit dsDNA high-sensitivity assay kit (Invitrogen, USA) (7, 9). Pooled libraries were loaded onto NovaSeq 6000 reagent cartridges, and paired-end short-reads (2 × 150 bp) were performed using the NovaSeq 6000 sequencer (Illumina, USA). Trimmomatic v.0.39 was used to remove low-quality reads (10). Draft genome sequences were assembled from trimmed and filtered reads using SPAdes v.1.3.15.5 (11), and the quality of the assemblies was evaluated using FASTQC v0.12.1 (12). The genomes were submitted to GenBank and annotated using the NCBI Prokaryotic Genome Annotation Pipeline (PGAP v.6.4) (13). Bioinformatics software available at the Center for Genomic Epidemiology (https://www.genomicepidemiology.org/) was used to identify acquired resistance genes (ResFinder v.4.1) (14) and potential pathogenicity (PathogenFinder v.1.1) (15). Default parameters were used for all software.

The isolates were identified as *Serratia* and *Enterobacter* species, showed resistance to important antibiotics, and carried acquired ABR genes (Table 1). This work highlighted the potential role of imported foods in the dissemination of antimicrobial resistance between countries.

**TABLE 1:** WGS analysis of *Serratia* spp. and *Enterobacter* sp. isolated from imported fresh produce in Georgia, USA

Sample ID	Fresh produce type	Country of origin	Retail store location (city in Georgia)	NCBI taxonomy	Genome size (bp)	No. of contigs	N_50_ (bp)	L_50_	Genome coverage (×)	GC (%)	Antibiotic resistance phenotypes^*a*^	Acquired ABR genes detected by ResFinder v.4.1^*b*^	Probability of being a human pathogen by PathogenFinder v.1.1^*c*^	SRA accession no.	GenBank accession no.	Genbank assembly accession number
**IPG4**	Leek	Mexico	Locus Grove	*Serratia ureilytica*	5,231,225	86	282,921	6	100	59.9	R: PEN-AMP-AMC-STR-TET-ERY S: FEP-CTX-CFM-DOR-IPM-MEM-GEN-KAN-CIP-NOR-SXT-CHL	*bla*_SST-1_; *tet(41*); *aac(6’)-Ic*	Yes (0.743)	SRR26787174	NZ_JAQOMO01	GCF_028621375.1
**IPG30**	Tomatillos	Mexico	Atlanta	*Serratia ureilytica*	5,084,533	85	182,050	10	100	59.9	R: PEN-AMP-AMC-STR-TET-ERY I: IPM S: FEP-CTX-CFM-DOR-MEM-GEN-KAN-CIP-NOR-SXT-CHL	*bla*_SST-1_; *tet(41*); *aac(6’)-Ic*	Yes (0.741)	SRR26787173	NZ_JAQOMM01	GCF_028620855.1
**IPG43**	Jicama root	Mexico	Atlanta	*Serratia marcescens*	5,101,936	40	512,362	2	100	59.8	R: PEN-AMP-AMC-STR-TET-ERY I: IPM S: FEP-CTX-CFM-DOR-MEM-GEN-KAN-CIP-NOR-SXT-CHL	*bla*_SST-1_; *tet(41*); *aac(6’)-Ic*	Yes (0.706)	SRR26787166	NZ_JARBGN01	GCF_028885965.1
**IPG66**	Asparagus	Peru	Locus Grove	*Serratia bockelmannii*	5,284,652	96	319,043	6	100	59.8	R: PEN-AMP-AMC- TET-ERY I: IPM-CHL S: FEP-CTX-CFM-DOR-MEM-GEN-KAN-STR-CIP-NOR-SXT-CHL	*bla*_SRT-2_; *tet(41*); *aac(6’)-Ic*	Yes (0.736)	SRR26787165	NZ_JAQOMI01	GCF_028616475.1
**IPG69**	Asparagus	Peru	Locus Grove	*Enterobacter bugandensis*	5,232,001	126	518,906	4	100	55.9	R: PEN-AMP-AMC- CFM-ERY-FOS I: TET S: FEP-CTX-DOR-IPM-MEM-GEN-KAN-STR-CIP-NOR-SXT-CHL	*fosA*	Yes (0.798)	SRR26787164	NZ_JAQMZS01	GCF_028616645.1
**IPG79**	Jalapeno pepper	Mexico	Griffin	*Serratia marcescens*	5,094,968	43	316,227	4	100	60	R: PEN-AMP-AMC-TET-ERY I: CFM-IPM S: FEP-CTX-DOR-MEM-GEN-KAN-STR-CIP-NOR-SXT-CHL	*bla*_SST-1_; *tet(41*); *aac(6’)-Ic*	Yes (0.716)	SRR26787163	NZ_JAQOMC01	GCF_028616965.1

^*a*^ The Kirby-Bauer disk diffusion test was used to determine the phenotypic antibiotic resistance profiles of the isolates according to the Clinical and Laboratory Standards Institute (CLSI) guidelines (5). R, resistance; I, intermediate resistance; S, susceptibility; PEN, penicillin; AMP, ampicillin; AMC, amoxicillin-clavulanic acid; FEP, cefepime; CTX, cefotaxime; CFM, cefixime; DOR, doripenem; IPM, imipenem; MEM, meropenem; GEN, gentamicin; KAN, kanamycin; STR, streptomycin; TET, tetracycline; CIP, ciprofloxacin; NOR, norfloxacin; SXT, trimethoprim-sulfamethoxazole; CHL, chloramphenicol, FOS, fosfomycin; ERY, erythromycin.

^*b*^ Acquired antibiotic resistance genes were detected using Resfinder v4.1 at default settings [threshold of %ID (90%) and minimum length (60%)].

^*c*^ Predicted probability of being a pathogen was determined using PathogenFinder v1.1. The probability of being a human pathogen is indicated in parentheses.

## Data Availability

The raw sequences and the assembled genome sequences for the analyzed strains were deposited in GenBank. The accession numbers for all the sequences are listed in Table 1. The annotated and assembled genome files are also available on Figshare: https://figshare.com/articles/dataset/Annotated_Assembled_Genomes_-_Georgia_USA/25341754
